# Cows are highly motivated to access a grooming substrate

**DOI:** 10.1098/rsbl.2018.0303

**Published:** 2018-08-08

**Authors:** Emilie McConnachie, Anne Marieke C. Smid, Alexander J. Thompson, Daniel M. Weary, Marek A. Gaworski, Marina A. G. von Keyserlingk

**Affiliations:** 1 Animal Welfare Program, University of British Columbia, 2357 Main Mall, Vancouver, British Columbia, Canada V6T1Z6; 2 Department of Production Management and Engineering, Warsaw University of Life Sciences, Nowoursynowska 166, 02-787 Warsaw, Poland

**Keywords:** animal behaviour, preference, animal welfare, push gate, enrichment

## Abstract

In natural environments, cattle use trees and other abrasive surfaces to scratch and groom themselves. Modern indoor dairy cattle housing systems often lack appropriate grooming substrates, restricting the animals' ability to groom. We assessed the motivation of dairy cows to access an automated mechanical brush, a grooming resource that can be implemented in indoor cattle housing systems. Cows were trained to push a weighted gate to access either fresh feed (positive control), a mechanical brush or the same space without a brush (negative control). Weight on the gate was gradually increased until all cows failed to open it. The weight each cow was willing to push to access each resource was assessed using the Kaplan–Meier survival analysis. Despite differences in methodology used to obtain data on motivation to access feed and the brush, the outcomes were very similar; cows worked as hard for access to fresh feed and the brush (*p* = 0.94) and less hard for access to the empty space (compared with fresh feed: *p* < 0.01; brush: *p* < 0.02). These results indicate that cows are highly motivated to access a mechanical brush and that it is an important resource for cows.

## 1. Introduction

Grooming helps animals remove dirt, parasites and other contaminants from skin and hair [1] and may positively influence the animals’ affective state [2,3]. Cows can groom themselves (by licking) and participate in allogrooming with herd mates and when kept in naturalistic environments, cows can use tree bark and other abrasive surfaces to scratch areas of the body that are otherwise difficult to reach. When kept indoors, cows may lack access to suitable grooming substrates that allow them to reach otherwise unreachable body areas. Some dairy farms provide access to automated mechanical brushes, providing cows a way to express grooming behaviour [4]. In certain countries, such as Denmark, providing cows with access to resources that promote coat care, such as mechanical brushes, is mandatory [5]. Cattle with access to mechanical brushes are cleaner and spend about fivefold more time grooming compared with when brushes are not available [6], suggesting that these brushes are important to the cow.

Motivation testing provides a method to assess the importance of a specific resource to an animal. The harder an animal is willing to work to gain access to the resource, the more important the resource is hypothesized to be to the animal. One method of measuring motivation is to train animals to push open a weighted gate to access a resource and then increase the weight over time, thereby increasing the work that must be performed to access the resource (e.g. [7–9]). Willingness to work for a resource of interest can then be compared with willingness to work for other resources, allowing inferences regarding their relative importance to the animal.

The aim of this study was to assess the motivation of dairy cows to access a mechanical brush by comparing this with their motivation to access fresh feed (as a positive control; food is a necessity, so cows would presumably have a maximal motivation for it, allowing this treatment to act as a motivation ‘yardstick’ (e.g. [10–12])) and the space the brush was in, without the brush (denoted empty space; a negative control). We hypothesized that motivation to access the mechanical brush would be lower than that for fresh feed, but higher than that for the empty space.

## 2. Material and methods

### (a) Habituation

Ten healthy, pregnant, lactating Holstein cows were used in this experiment (mean ± s.d. days in milk: 207 ± 24). Animals were given 5 days for social interactions to stabilize [13] and to habituate to the experimental pen and mechanical brush. See electronic supplementary material, S1 for more information on the subjects and housing.

### (b) Training

All animals were trained to push open a weighted gate from a closed position. To be included in the experiment, cows needed to be able to open the push gate with 7 kg of weight attached to it without a trainer present. See electronic supplementary material, S1 for more information on training and the push gate apparatus.

### (c) Testing

Motivation testing took place in four consecutive treatments: (i) mechanical brush I, (ii) fresh feed (total mixed ration (TMR)), (iii) empty space and (iv) mechanical brush II. Cows were not tested in a balanced order because of the constraints of having access to only a single push gate and working within the day-to-day operations of a dairy farm. However, we did partially test for an order effect within the framework of sequential treatments by testing for the mechanical brush treatment twice, once at the beginning of the experiment and once at the end of the experiment.

The push gate restricted access to the resource in each treatment and weight was added until no cow pushed open the gate. In this way, we determined the maximum price paid by each cow per treatment. For both brush treatments and the empty space treatment, animals had continuous access to the push gate, and weights were added every 3 days. In measuring motivation to access fresh TMR, these methods had to be altered; to follow the existing methods for that treatment would have required long periods of food restriction. Therefore, the fresh feed treatment consisted of daily testing where the cows were individually brought into the experimental pen and allowed 15 min to push open the push gate to access fresh feed. If a cow succeeded in pushing the push gate to get access to the feed, weights were added to the subsequent day's testing. For this treatment, the cows were deprived of feed for approximately 1.5 h and fresh feed for approximately 15 h before testing. Between treatments, all cows were subjected to three training sessions over a 3-day period. The final (mechanical brush II) treatment was cut short by 3 days, in comparison with mechanical brush I, as the cows were approaching their expected calving dates (the experiment ended 49 ± 36 days before expected calving date).

See electronic supplementary material, S1 for more information on Material and methods.

## 3. Results

**Figure 1.**
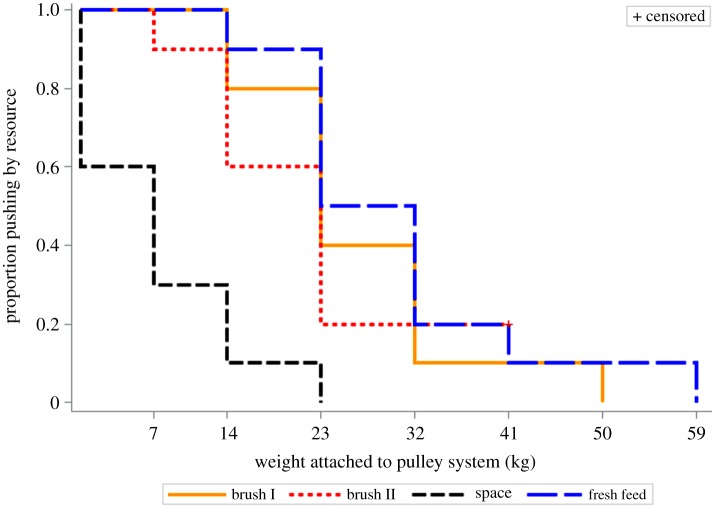
Survival plot of cow (*n* = 10) willingness to work for access to a mechanical brush, empty space and fresh feed. Cows pushed a similar weight for access to the brush and for access to fresh feed, as indicated by the weight on the pulley system. Weight pushed was lower for empty space. Motivation for these resources was tested in sequential treatments: (i) mechanical brush I, (ii) fresh feed, (iii) empty space and (iv) mechanical brush II. Motivation for the mechanical brush was tested twice in order to partially test for an order effect. For the brush and space treatments, cows had continuous access to the push gate for 3 days. Feed access could not be constantly restricted due to animal welfare concerns and thus methods had to be altered for the feed treatment. However, motivation for food (i.e. hunger) is easier to manipulate than motivation to groom as there is more predictable, consistent behaviour evoked by food than a brush. Therefore, for this treatment, cows were deprived of feed for approximately 1.5 h and fresh feed for approximately 15 h after which they were individually subjected to a 15 min testing session, during which time cows had the opportunity to push open the push gate to access food. Data from mechanical brush II were right-censored to account for the early end of treatment. (Online version in colour.)

No difference was found in the weight pushed between the two treatments testing motivation to access the mechanical brush (*p* = 1.00; figure 1). In addition, there was no difference in weight pushed for access to the mechanical brush I and fresh feed (*p* = 0.94) or in weight pushed for mechanical brush II and fresh feed (*p* = 0.85). However, cows pushed less weight to access the empty space than to access the brush in treatment I (*p* < 0.02), the brush in treatment II (*p* < 0.02) and fresh feed (*p* < 0.01).

See electronic supplementary material, S1 and S2 for more information on statistical analysis, and electronic supplementary material, S3 for the datasets supporting this article.

## 4. Discussion

Dairy cows were similarly motivated to access a mechanical brush and to access fresh feed; despite differences in methodology of data collection between the brush and feed treatment, this result suggests that the brush is a valued resource for cows. The brush facilitates grooming, a highly conserved natural behaviour across species [1]. Grooming provides animals with a way to cope with stressors [2] and has been shown to increase oxytocin levels in rats [3]. By having access to the mechanical brush, cows were better able to groom, possibly increasing positive affect [14].

Motivation to access the empty space was lower than that to access either fresh feed or the brush, indicating that cows were motivated to access the mechanical brush during the brush treatments. That said, three cows did work to access the empty alley, and all cows accessed the empty alley during the interval periods between treatments, so the empty alley was not novel to them. Visiting the empty alley may be viewed as a type of exploratory behaviour; such exploration is consistent with the cows being motivated to learn about their environment [15,16].

Our results may have been impacted by a ceiling effect, meaning that cows may have been physically unable to push open the gate past a certain weight. At least some of the cows' unsuccessful attempts to open the gate may have been due to the animal physically being unable to perform the task, rather than her being unmotivated. Indeed, we observed cows continuing to attempt to push open the gate after they had reached their experimental failure weight. Additionally, the weighted gate pressed on the side of the cow as she passed through the gate. The cows were pregnant, which may have made this passage more uncomfortable for them. Future work should examine other types of motivational tests or test cows that are not pregnant. Other types of experimental designs for motivation tests that may be less susceptible to ceiling effects may include manipulating walking distance [17] or lever pressing [18]. Increasing distance from food location has been shown to reduce brush usage in cows, suggesting that this may be a promising avenue to explore [19]. It may also be interesting to explore motivation to access a brush or other grooming substrates in other types of dairy farms, including pasture-based systems.

## 5. Conclusion

Cows were similarly motivated to access a mechanical brush and to access fresh feed and more motivated to access both of these resources than to access an empty space. These results indicate that the automated mechanical brush is an important resource for cows.

## Supplementary Material

Detailed Methods and Analysis

## Supplementary Material

Experimental Data

## Supplementary Material

SAS Code

## Data accessibility

The datasets and code supporting this article have been uploaded as part of the electronic supplementary material.

## References

[1] Spruijt BM, van Hooff JA, Gispen WH 1992 Ethology and neurobiology of grooming behavior. Physiol. Rev. 72, 825–852. (10.1152/physrev.1992.72.3.825) 1320764

[2] Newby NC, Duffield TF, Pearl DL, Leslie KE, LeBlanc SJ, von Keyserlingk MA 2013 Short communication: use of a mechanical brush by Holstein dairy cattle around parturition. J. Dairy Sci. 96, 2339–2344. (10.3168/jds.2012-6016) 23462171

[3] Stock S, Uvnas-Moberg K 1988 Increased plasma levels of oxytocin in response to afferent electrical stimulation of the sciatic and vagal nerves and in response to touch and pinch in anaesthetized rats. Acta Physiol. Scand. 132, 29–34. (10.1111/j.1748-1716.1988.tb08294.x) 3223304

[4] Mandel R, Whay HR, Klement E, Nicol CJ 2016 Invited review: environmental enrichment of dairy cows and calves in indoor housing. J. Dairy Sci. 99, 1695–1715. (10.3168/jds.2015-9875) 26774729

[5] Jensen SM (ed.). 2013 *World class cattle production*. Aarhus, Denmark: Danish Agriculture & Food Council, Cattle. https://www.seges.dk/en/fagomraader/kvaeg/~/media/C54CC7E0B12D4FF395D2E4469A8F061A.ashx.

[6] DeVries TJ, Vankova M, Veira DM, von Keyserlingk MAG 2007 Short communication: usage of mechanical brushes by lactating dairy cows. J. Dairy Sci. 90, 2241–2245. (10.3168/jds.2006-648) 17430923

[7] Olsson IAS, Keeling LJ 2002 The push-door for measuring motivation in hens: laying hens are motivated to perch at night. Anim. Welf. 11, 11–19.

[8] Petherick JC, Rutter SM 1990 Quantifying motivation using a computer-controlled push-door. Appl. Anim. Behav. Sci. 27, 159–167. (10.1016/0168-1591(90)90015-6)

[9] Tucker CB, Munksgaard L, Mintline EM, Jensen MB 2018 Use of a pneumatic push gate to measure dairy cattle motivation to lie down in a deep-bedded area. Appl. Anim. Behav. Sci. 201, 15–24. (10.1016/j.applanim.2017.12.018)

[10] von Keyserlingk MAG, Cestari AA, Franks B, Fregonesi JA, Weary DM 2017 Dairy cows value access to pasture as highly as fresh feed. Sci. Rep. 7, 744953 (10.1038/srep44953) PMC5362966 28332567

[11] DeVries TJ, von Keyserlingk MAG, Beauchemin KA 2003 Short communication: diurnal feeding pattern of lactating dairy cows. J. Dairy Sci. 86, 4079–4082. (10.3168/jds.S0022-0302(03)74020-X) 14740847

[12] Dawkins MS 1988 Behavioural deprivation: a central problem in animal welfare. Appl. Anim. Behav. Sci. 20, 209–225. (10.1016/0168-1591(88)90047-0)

[13] von Keyserlingk MAG, Olenick D, Weary DM 2008 Acute behavioral effects of regrouping dairy cows. J. Dairy Sci. 91, 1011–1016. (10.3168/jds.2007-0532) 18292257

[14] Fraser D, Weary DM, Pajor EA, Milligan BN 1997 A scientific conception of animal welfare that reflects ethical concerns. Anim. Welf. 6, 187–205.

[15] Wood-Gush DGM, Vestergaard K 1989 Exploratory behavior and the welfare of intensively kept animals. J Agric. Ethics 2, 161–169. (10.1007/BF01826929)

[16] Boissy A et al. 2007 Assessment of positive emotions in animals to improve their welfare. Phys. Behav. 92, 375–397. (10.1016/j.physbeh.2007.02.003) 17428510

[17] Verbeek E, Waas JR, McLeay L, Matthews LR 2011 Measurement of feeding motivation in sheep and the effects of food restriction. Appl. Anim. Behav. Sci. 132, 121–130. (10.1016/j.applanim.2011.03.014)

[18] Cooper MD, Arney DR, Phillips CJC 2010 The motivation of high- and low-yielding dairy cows to obtain supplementary concentrate feed. J. Vet. Behav. Clin. Appl. Res. 5, 75–81. (10.1016/j.jveb.2009.09.045)

[19] Mandel R, Whay HR, Nicol CJ, Klement E 2013 The effect of food location, heat load, and intrusive medical procedures on brushing activity in dairy cows. J. Dairy Sci. 96, 6506–6513. (10.3168/jds.2013-6941) 23958014

