# Supplementary material for: Cows are highly motivated to access a grooming substrate
Source: Biol Lett. 2018 Aug 8;14(8):20180303. doi: 10.1098/rsbl.2018.0303 (PMC6127119; doi:10.1098/rsbl.2018.0303)
Supplement: Detailed Methods and Analysis [file rsbl20180303supp1.docx]

**Detailed Methods and Analysis (Electronic Supplementary Materials 1) for:**

**Cows are highly motivated to access a grooming substrate**

Emilie McConnachie, Anne-Marieke C. Smid, Alexander J. Thompson, Daniel M. Weary, Marek A. Gaworski, and Marina A. G. von Keyserlingk

Corresponding author email: [marina.vonkeyserlingk@ubc.ca](mailto:marina.vonkeyserlingk@ubc.ca)

***Cows and housing***

This experiment took place at the University of British Columbia’s Dairy Education and Research Centre in Agassiz, B.C., Canada from May-September 2016. Twelve Holstein cows (mean ± SD days in milk: 207 ± 23.7; mean ± SD age: 4 ± 0.74) were initially selected for this study, a number similar to other animal motivation studies (e.g. 1–3). All cows were in mid to late lactation and pregnant (to avoid animals in heat), had a body condition score no lower than 2.5 on a scale of 1-5 with quarter-point increments (4), no known diseases, and a gait score of 1-3 on a scale of 1-5 (5). Prior experience with a mechanical brush and familiarity with one another were not considered during sample selection. Cows were each given a unique symbol made with human hair dye to facilitate individual recognition on the video recordings.

Cows were housed in a free-stall pen containing 24 lying stalls deep bedded with sand (115 cm wide x 205 cm long with a neck rail placed 125 cm above the sand), 12 electronic feeding bins, and 2 electronic water bins (Insentec, Marknesse, Holland). To facilitate training and testing of motivation for fresh feed, we used an adjacent pen, denoted the waiting pen, fitted with 12 lying stalls deep bedded with sand (Figure 1A). Cows were fed a total mixed ration (TMR) consisting of 33% corn silage, 48% concentrate mash, 14% grass silage, and 5% alfalfa hay. Fresh TMR was provided twice daily at approximately 0645 and 1645 h (orts were removed approximately 15 minutes prior to afternoon feeding) and cows were moved to the milk parlour for milking twice daily at approximately 0700 and 1700 h.

Two one-way push gates were used in this study. One was the weighted, experimental push gate (Figure 2) that cows were required to push to access the tested resource. The other gate (referred to as the return gate) was positioned so that cows could return from the back alley (Figure 1B) and thus was only used when testing the mechanical brush and empty space treatments. The return gate was unweighted but some tension was provided using bungee cords. Both the push gate and return gate were used in the training and interval periods (discussed in following paragraphs).

Four video cameras (Panasonic WV-CP310, Osaka, Japan) and red lights (100 W) were mounted above the home pen to allow for 24 hr/d video surveillance of the pen. A mechanical brush (Lely Luna cow brush, Maassluis, The Netherlands) was placed at a height of 150 cm in the back alley (Figure 1A).

***Push gate apparatus***

A pulley system was used to load the push gate with weights (designed by M.A.G.; Figure 1). To better understand the energetic cost to access the resources tested, a force plate was used on the front of the push gate (provided by M.A.G.; developed by Jacek Domitrz at ZEPWN, Marki, Poland) to estimate the force (measured in kilogram-force [kgf]) required to open the gate when different weights were added to the pulley system. We chose weights to provide approximate 20 kgf increments in the force required to open the gate (Table 1).

***Training and interval period***

During training, each cow was taught how to push open the push gate. All 12 cows were trained once a day, with six randomly selected animals trained in the morning and the remaining six trained in the afternoon (the application “Random Picker” by Byroid Apps was used for random selection). Each six cow training session lasted approximately 90 minutes. The gate was left in the open position outside of training times, allowing animals’ free access to the mechanical brush.

For every training session, all cows were placed in the waiting pen, without access to TMR and water but with access to all 12 lying stalls. Animals were randomly selected (“Random Picker” app used for selection) one at a time without replacement to be trained and were brought into the home pen where they would practice pushing open the push gate and the return gate by completing a loop between the main and back alley four times per session (Figure 1B). Initially, individual cows were lead through open gates to demonstrate the location of the TMR reward and to habituate them to the loop. Following each successful loop, the gate was closed by 15° until it was set at a fully closed position. Following each failed attempt, the gate was opened by 15° relative to the previous position. If the cow successfully opened the push gate, her behavior was positively reinforced with access to TMR for 30 seconds. Each cow completed four loops per session.

When all cows had successfully pushed open the fully closed push gate, a 2.3 kg weight was added to the pulley system. When a cow successfully opened the gate, another 2.3 kg was added. When a cow failed an attempt to open the gate, weight was decreased by 2.3 kg on the next attempt. Once a cow met the 7 kg requirement, she continued training with this weight, but for only two loops per session, until all cows met the 7 kg requirement. The 7 kg threshold was chosen because this ensured that the gate would swing closed after being opened. The training phase ended when all animals opened the push gate with 7 kg for 3 successive days. Training took approximately 3 weeks to complete.

After training, the cows were given 3 days where the push gate was closed with bungee cords rather than with weights. During this period, animals could only access the mechanical brush by opening the push gate. The aim of this interval period was to ensure that the animals were capable of pushing the push gate from the closed position without a trainer present. Three days was chosen because a pilot study showed that all cattle used the brush at least once every 3 days when free access was given. To ensure that we only included cows that had successfully learned how to open the gate and continued to do so outside of a training session, a two-part learning criterion was set. Cows we required to 1) push open the gate from a closed position with 7 kg of weight during training, and 2) push open the gate during the interval period (without an experimenter present) to meet this criteria and be included in the study.

***Treatment details***

After training was complete, the four treatments were tested successively with the same group of 10 cows (two cows were excluded from analysis; see “Statistical Analysis” below for more information) throughout (Figure 3). Cows were not tested in a balanced manner because of the constraints of having access to only a single push gate and working within the day to day operations of a dairy farm. However, we did partially test for an order effect within the framework of sequential treatments by testing for the mechanical brush treatment twice, once at the beginning of the experiment and once at the end of the experiment to capture any possible effect of progressive pregnancy and order effects within the timeline of our experiment. The first day of the first brush treatment and the first day of the second brush treatment were separated by 58 days, and no difference in motivation to access the brush between treatments was observed.

***Treatments***

1. Access to the mechanical brush (I and II)

For the duration of this treatment, animals were housed in their home pen and were required to push the weighted gate from a closed position to access the mechanical brush. The trial started with 7 kg of weight attached on the pulley system; 3 days later another 7 kg of weight was added and every 3 days after, 9 kg of weight was added. When a weight was reached for which no cow successfully opened the gate over the course of 3 days, a final 9 kg was added for 3 days and if no cow successfully pushed this weight, testing ended. Thus, in every treatment all cows reached a point at which they were either unwilling or unable to open the gate, possibly creating a negative experience and association with the gate for the cows. To minimize this negative experience and association, cows were provided 6 consecutive days after the end of each treatment in which they were re-trained and transitioned to the next treatment using the un-weighted gate (Figure 3). This approach was deemed successful, as no difference in motivation existed between the two mechanical brush treatments (i.e. the treatments tested first and last of the sequence).

Using continuous video observations we recorded when the cows visited the back alley, and for each visit, the amount of time each cow spent in the back alley, the amount of time she interacted with the brush (interaction was defined as when the cow touched the brush with any part of her body, causing the mechanical brush to start rotating until the point when the cow ended contact with the brush), and the number of brush interactions per alley visit.

2. Access to fresh TMR

To measure motivation to access fresh TMR, the push gate was moved to the platform between the main and feed alley (Figure 1C). Animals were then moved into the waiting pen daily at 0630 h, where a locked gate blocked access to the feed alley of this pen. Cows were then milked, returning to the waiting pen at approximately 0800 h, at which time testing began. This way, animals did not have access to the TMR for approximately 1.5 h before testing and were last given *fresh* TMR the previous evening.

Cows were brought individually into the home pen and given 15 min to open the gate. The cow was gently guided halfway up the main alley but allowed to approach the gate unguided. If the cow came back down the main alley past the halfway point, she was gently turned around. This process was repeated until either the cow successfully pushed the gate or the time of testing exceeded 15 min. If a cow successfully opened the gate, she was allowed access to the fresh TMR for 30 s after which she was returned to the feed alley of the waiting pen. If she did not succeed, she was returned to the main alley of the waiting pen without access to TMR until the day’s session was over. If a cow failed her session, the weight on the push gate remained the same the following day. If a cow successfully pushed open the gate, the weight was increased in the next day’s session, in the same increments as the mechanical brush treatments (1x7 kg increase then 9 kg increases until the end of the treatment). Testing continued daily for each individual cow until all cows failed 3 sessions in a row. Cows who had failed 3 sessions were still moved to the waiting pen for the following sessions, but only cows who had not failed 3 sessions underwent the sessions’ testing.

3. Access to empty space

For this treatment, the push gate was returned to the platform between the main alley and back alley and the mechanical brush was removed, leaving the back alley empty. The same methods used to test motivation to access the mechanical brush in the first treatment were followed to test motivation to access this empty space.

***Differences between feed and brush methodology***

We fully acknowledge that there are methodological differences between feed and brush treatments (see 6 for a similar experiment) and we thank an anonymous reviewer for encouraging us to explain these differences further.

A key idea to our methodology is that it is easier to manipulate motivation for food (i.e. hunger) than the motivation to groom. There are differences in these behaviors that contribute to this phenomenon; for example, during a pilot study, we noted that certain cows used the brush every day, others used it at very specific times, and others used it once every three days. However, all cows ate immediately after fresh feed delivery in both the morning and afternoon. From this, it is clear that there is more predictable, consistent behavior evoked by food than the brush.

Another study found that delivery of fresh feed had a greater stimulating effect on cow feeding than return from milking (7). This study exemplified how we could use fresh feed to manipulate feeding motivation further. In fact, we could arguably increase motivation for access to fresh feed to a more extreme level if we combined both feed deprivation alongside feed delivery. Ultimately, this combination created what we considered to be our motivation “yardstick”; motivation to feed would be at a peak under these conditions, but within the framework of Canadian Council on Animal Care (8) regulations on animal treatment.

Our goal was to evaluate the motivation for feed by use of a push-gate under these more extreme conditions. We prioritized capturing the full extent of brush motivation alongside the full extent of motivation for fresh feed, recognizing that there are differences in methodology.

***Statistical Analysis***

Twelve healthy cows were initially included in the study. However, one cow did not meet the learning criteria and was thus excluded from the study. To minimize disruption of the test group’s social structure, this cow remained in the pen with the experimental cows throughout the study. Another cow had an abortion early on in the study, so her data was also excluded; this cow was moved to a hospital pen to allow for better treatment. Thus, the final sample size used was 10 cows.

Data were analyzed using SAS (version 9.4, SAS Institute, Institute Inc., Cary, NC) treating cow (n = 10) as the experimental unit. Normality of data was analyzed using the PROC UNIVARIATE procedure.

Using the PROC LIFETEST procedure, a Kaplan-Meier survival analysis was conducted to estimate the survival curves for each of the resources. Data from mechanical brush II was right-censored to account for the early end of treatment. As all treatments were included in the same model, Tukey-Kramer adjusted p-values were used.

For all analyses, significance was accepted when *P* < 0.05.

**References**

1. Jensen MB, Munksgaard L, Pedersen LJ, Ladewig J, Matthews L. Prior deprivation and reward duration affect the demand function for rest in dairy heifers. Appl Anim Behav Sci. 2004;88(1–2):1–11.

2. Pedersen LJ, Jensen MB, Hansen SW, Munksgaard L, Ladewig J, Matthews L. Social isolation affects the motivation to work for food and straw in pigs as measured by operant conditioning techniques. Appl Anim Behav Sci. 2002;77(4):295–309.

3. Cooper JJ, Mason GJ. The use of operant technology to measure behavioral priorities in captive animals. Behav Res Methods Instrum Comput. 2001;33(3):427–34.

4. Edmonson AJ, Lean IJ, Weaver LD, Farver T, Webster G. A Body Condition Scoring Chart for Holstein Dairy Cows. J Dairy Sci. 1989;72(1):68–78.

5. Flower FC, Weary DM. Effect of Hoof Pathologies on Subjective Assessments of Dairy Cow Gait. J Dairy Sci. 2006;89(1):139–46.

6. von Keyserlingk MAG, Cestari AA, Franks B, Fregonesi JA, Weary DM. Dairy cows value access to pasture as highly as fresh feed. Sci Rep. 2017;744953:DOI: 10.1038/srep44953.

7. DeVries TJ, von Keyserlingk MAG. Time of Feed Delivery Affects the Feeding and Lying Patterns of Dairy Cows. J Dairy Sci. 2005;88(2):625–31.

8. Canadian Council on Animal Care. CCAC guidelines on: the care and use of farm animals in research. 2009.


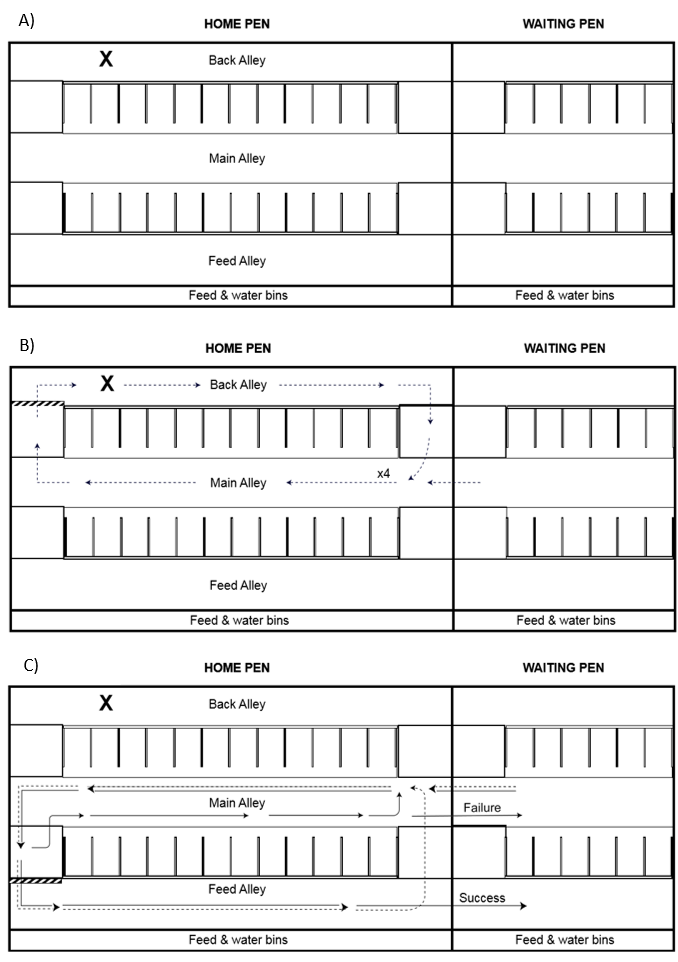


**Figure 1. A)** Overview of the experimental set-up. The mechanical brush is represented by the “X” in the back alley. **B)** Training, mechanical brush treatments, and empty space treatment set-up. Dotted lines denote the training loop. During training only, a bucket containing the total mixed ration (TMR) was placed in the back alley by the mechanical brush, ropes were used to restrict access to lying stalls, and the feed alley was blocked. Between the back alley and main alley, the one-way weighted push gate is represented by the thick, striped bar and the one-way return gate is represented by the thinner, solid bar. For the space treatment, the mechanical brush was removed. **C)** TMR treatment set-up. The one-way weighted push gate was moved to an identical raised platform across the main alley to restrict access to the feed alley during testing. Dotted lines denote the training loop. Solid lines represent cow movement during testing. During training and testing, ropes were used to prevent access to the lying stalls and back alley. A gate was closed in the waiting pen between the main alley and feed alley to separate the successful cows from the cows who failed.

**Table 1.** Estimated force (±5 kgf; as measured by the force plate) required to open the gate by manually pushing on the center of the force plate and holding the gate open 25 cm.

| **Weight** | **Force** |
| --- | --- |
| 7 kg | 20 kgf |
| 14 kg | 40 kgf |
| 23 kg | 60 kgf |
| 32 kg | 80 kgf |
| 41 kg | 100 kgf |
| 50 kg | 120 kgf |
| 59 kg | 140 kgf |


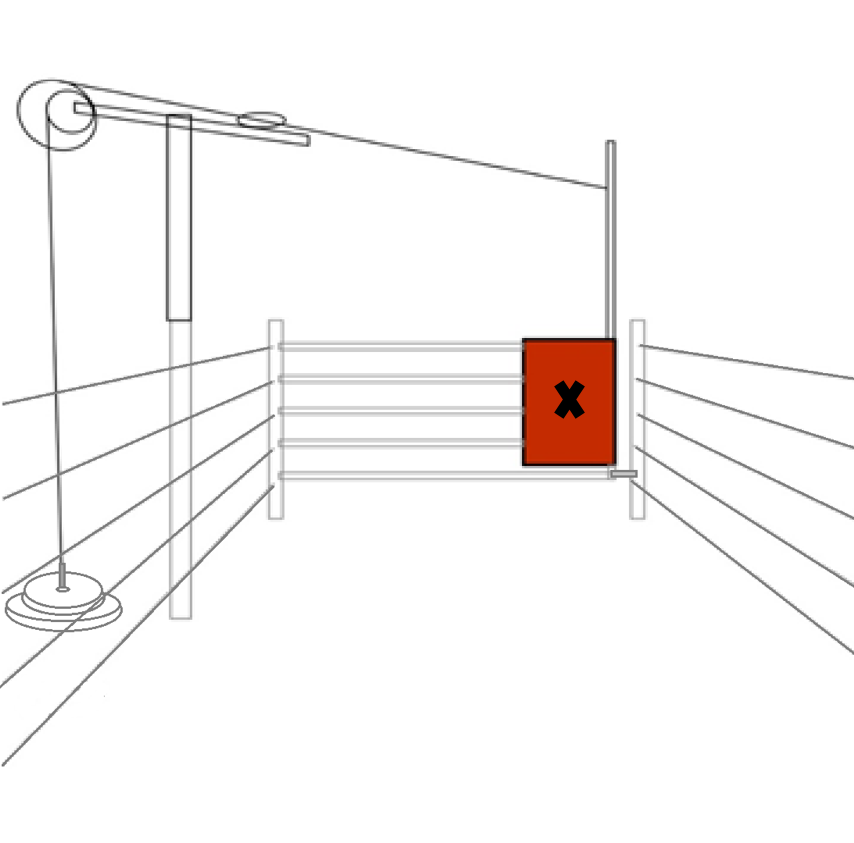


**Figure 2.** Schematic of the weighted push gate. A red force plate was positioned on the gate to help target the cows’ pushing behavior. The “X” in the center of the force plate indicates where it was pushed to estimate kgf. Barbell weights were attached to the gate via a pulley mechanism.


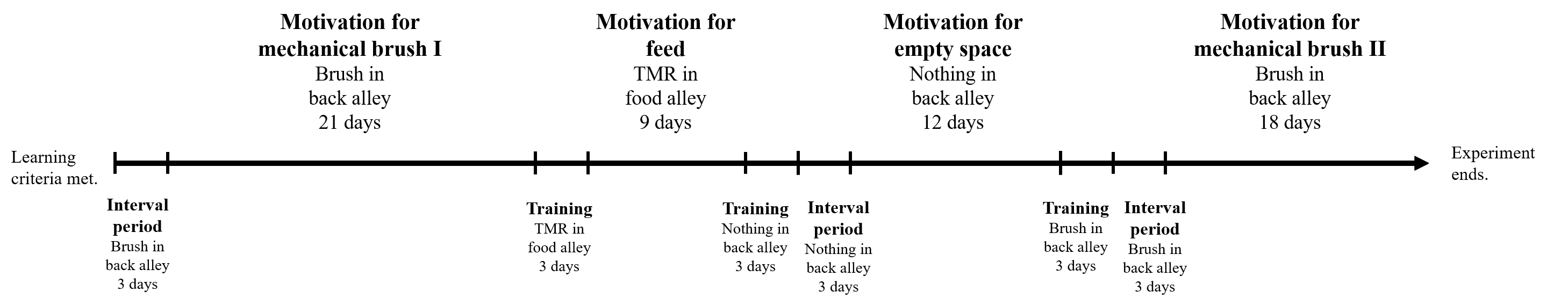


**Figure 3.** Timeline of experimental treatments. Tested resource, resource location, and length of phase noted. Between each treatment, cows underwent a brief training and/or interval period to ensure they still knew how to open the gate and didn’t associate a closed gate with the previous tested resource. Training for each cow consisted of 1 session/d comprised of 3 pen laps with 7 kg of weight attached to the push gate. During this period, the gate was left open outside of training sessions. After training, a 3 day interval period took place where the push gate was closed with bungee cords, restricting access to the tested resource 24 hr/d. This period did not occur before the feed trials as we did not want to restrict access to feed for prolonged periods.
